# Implementation of a regional quality improvement collaborative to improve care of people living with opioid use disorder in a Canadian setting

**DOI:** 10.1186/s12913-019-4472-8

**Published:** 2019-09-14

**Authors:** Laura Beamish, Zach Sagorin, Cole Stanley, Krista English, Rana Garelnabi, Danielle Cousineau, Rolando Barrios, Jan Klimas

**Affiliations:** 1 British Columbia Centre for Excellence in HIV/AIDS (BC-CfE), St. Paul’s Hospital, 608-1081 Burrard Street, Vancouver, BC V6Z1Y6 Canada; 2 Vancouver Coastal Health, 520 W 6th Ave, Vancouver, BC V5Z4H5 Canada; 3 St. Paul’s Hospital, Providence Health Care, 608-1081 Burrard Street, Vancouver, BC V6Z1Y6 Canada; 4 British Columbia Centre on Substance Use, 400-1045 Howe St, Vancouver, BC V6Z2A9 Canada; 5 Faculty of Medicine, University of British Columbia, 2206 East Mall, Vancouver, BC V6T1Z3 Canada; 6 Department of Medicine, University of British Columbia, St. Paul’s Hospital, 608-1081 Burrard Street, Vancouver, BC V6Z1Y6 Canada; 7 School of Medicine, Health Sciences Centre, University College Dublin, Belfield, Dublin 4, Ireland

**Keywords:** Opioid use disorder, Opioid agonist therapy, Methadone, Suboxone, Buprenorphine/naloxone, Retention, Quality improvement, Primary care, Substance use disorder, Mental health

## Abstract

**Background:**

Although opioid agonist therapy is effective in treating opioid use disorders (OUD), retention in opioid agonist therapy is suboptimal, in part, due to quality of care issues. Therefore, we sought to describe the planning and implementation of a quality improvement initiative aimed at closing gaps in care for people living with OUD through changes to workflow and care processes in Vancouver, Canada.

**Methods:**

The Best-practice in Oral Opioid agoniSt Therapy (BOOST) Collaborative followed the Institute for Healthcare Improvement’s Breakthrough Series Collaborative methodology over 18-months. Teams participated in a series of activities and events to support implementing, measuring, and sharing best practices in OAT and OUD care. Teams were assigned monthly implementation scores to monitor their progress on meeting Collaborative aims and implementing changes.

**Results:**

Seventeen health care teams from a range of health care practices caring for a total of 4301 patients with a documented diagnosis of OUD, or suspected OUD based on electronic medical record chart data participated in the Collaborative. Teams followed the Breakthrough Series Collaborative methodology closely and reported monthly on a series of standardized process and outcome indicators. The majority of (59%) teams showed some improvement throughout the Collaborative as indicated by implementation scores.

**Conclusions:**

Descriptive data from the evaluation of this initiative illustrates its success. It provides further evidence to support the implementation of quality improvement interventions to close gaps in OUD care processes and treatment outcomes for people living with OUD. This system-level approach has been spread across British Columbia and could be used by other jurisdictions facing similar overdose crises.

## Background

Canada, much like the rest of North America, is in the midst of an overdose crisis. Since 2016, approximately 10,000 people have died as a result of opioid-related overdose, with the province of British Columbia (BC) reporting the highest rates of accidental opioid-related deaths [1]. The crisis in BC has highlighted significant gaps in care for people living with opioid use disorders (OUD).

Current Canadian clinical guidelines for the management of OUD recommend the use of opioid agonist therapies (OAT) as first line treatment [2]. A recent meta-analysis demonstrated that retention in OAT is associated with two to three times lower all-cause and overdose-related mortality in people with OUD [3]. A significant proportion of individuals with OUD reduce illicit opioid use, remain in treatment using appropriate doses of OAT, such as methadone and buprenorphine/naloxone [4].

Delivering appropriate care and treatment for OUD is a complex process that requires collaboration among the most responsible provider (MRP) and other care providers - from screening and diagnosis to treatment and follow-up [5, 6]. The term MRP (most responsible physician/practitioner/provider), generally refers to regulated healthcare professional, who has overall responsibility for directing and coordinating the care and management of a patient at a specific point in time. While typically referring to a physician, this may include a nurse practitioner or other healthcare professional [7]. Establishing effective and proactive systems of care within which OUD treatment can be delivered is essential for treatment to be successful and sustainable. The chronic care model (CCM) is cited as an effective organizing framework for improvement initiatives [8].

Increasingly, the healthcare community is employing quality improvement (QI) frameworks to promote system-level change and address gaps in practice [9]. The Breakthrough Series (BTS) Collaborative methodology, developed by the Institute for Healthcare Improvement (IHI), is a QI approach designed to help healthcare organizations systematically close the gaps between evidence and practice [10]. Although literature reported variable success of QI approaches [11], BTS has been successfully applied in other drug treatment settings in the United States [12–14], and for other chronic diseases in Canada, including diabetes, congestive heart failure and most recently HIV/AIDS [15–17]. In 2017, the BC Centre for Excellence in HIV/AIDS (BC-CfE)—a provincial resource for HIV/AIDS care, treatment, education, research and evidence-based policy development— and Vancouver Coastal Health (VCH) launched a BTS Collaborative, titled the best-practice in oral opioid agonist therapy, or the BOOST Collaborative. The initiative aimed to systematically implement, measure and share best-practices in oral opioid OAT and improve outcomes for people living with OUD in Vancouver, Canada. The current paper sought to describe the planning and implementation of a BTS Collaborative aimed at closing gaps in care for people living with OUD through changes to workflow and care processes.

### Context

British Columbia is Canada’s westernmost province and has a population of approximately 4.8 million [18]. In 2016, the province declared a state of public health emergency in response to the alarming increase in the rate of opioid-related overdose deaths [19]. BC is at the epicentre of the overdose crisis in Canada with over 4000 opioid-related overdose deaths since the declaration, with over 1100 of those deaths occurring in the Vancouver region [1]. The majority of opioid-related overdose deaths occur in men aged 30–39 and disproportionately affect Indigenous and/or First Nations people of BC^1^. Current data from the Office of the Provincial Health Officer shows poor OAT retention rates in Vancouver, with only 42% of people who start OAT retained at six months, with this dropping to 32% at 12 months [20]. The province has launched a number of services to address the crisis, including the scale-up of naloxone distribution, the expansion and establishment of supervised consumptions sites, expanded low barrier substance use disorder (SUD) care, and improved access to treatment such as OAT [21]. Although considerable work has been done to address the crisis, the death rate remains well above historical average [1]. BC Coroners Service data shows the majority of opioid-related overdose deaths are occurring in people not retained on OAT, highlighting a key gap in care [22, 23]. With increasing evidence that OAT can reduce overdose and all-cause mortality, Vancouver Coastal Health regional health authority the BC-CfE committed funding for a QI Collaborative to launch in September 2017. This pilot was called the Vancouver Best-practices in Oral Opioid agoniSt Therapy, or the BOOST, Collaborative.

### Health system organization

The health care system in BC is predominantly funded publicly through taxation and other revenue sources [23]. In the provincial government, health care is organized into the Ministry of Health and the newly formed Ministry of Mental Health and Addiction. These two Ministries are responsible for setting the strategic direction and priorities in the province [23]. Health care funding is directed from the Ministry of Health to five regional health authorities who deliver care in their respective regions. There are also two provincial health authorities responsible for specific programs and populations across the province [23]. In BC, pharmaceutical drugs are not publicly funded, however the Ministry of Health funds OAT (methadone, buprenorphine/naloxone, and slow release oral morphine) for populations who show both a medical and financial need [24].

The majority of care for people with OUD in Vancouver is delivered in interprofessional community health centres where physicians are remunerated at a government negotiated sessional rate [25], making them the focus of this intervention. In some cases, OUD care is provided in fee-for-service private practice settings or in hospital settings for a short duration of time (induction and stabilization) [26].

## Methods

### Intervention components

**Fig. 1:**
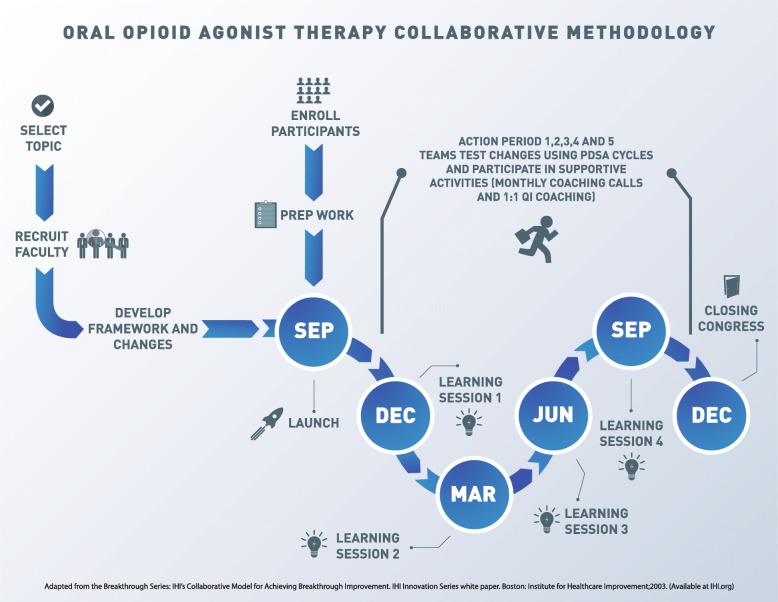
Oral Opioid Agonist Therapy Collaborative Methodology

The BOOST Collaborative followed the IHI’s BTS Collaborative methodology for 18-months from September 2017 to December 2018 (see Fig. 1). The overarching aim of the BOOST Collaborative was to provide equitable access to integrated, evidence-based care to help our population of clients with OUD achieve: 95% initiated on OAT; 95% retained in care for ≥3 months; and 50% average improvement in quality of life scores. The BC Centre for Excellence in HIV/AIDS—a provincial resource for HIV/AIDS care, treatment, education, research and evidence-based policy development—provided intervention leadership and coordination with QI support from the Practice Support Program, a program of the General Practice Services Committee at the Doctors of BC dedicated to in-practice QI coaching and support. The program was funded by Vancouver Coastal Health with in-kind contributions from the BC Centre for Excellence in HIV/AIDS. Delivery costs included learning sessions, educational webinars, and Collaborative staff time and travel. Vancouver Coastal Health covered the cost of staff participation and travel to learning sessions, including the required backfill.

#### Planning and team recruitment

In July 2017 a multi-stakeholder working group meeting was held to help shape the technical content of the BOOST Collaborative. The working group had representation from across programs and health disciplines in Vancouver Coastal Health along with representation from the community and people with lived experience. Directors from primary care, mental health and substance use programs in Vancouver Coastal Health were asked to identify programs that could participate in the Collaborative process, contribute to the shared aims, collect indicators and implement changes.

#### Launch and preparation

In August 2017, programs that were identified by leadership to participate, were invited to attend a 60-min informational webinar that aimed to introduce the purpose, aims and measurement strategy of the BOOST Collaborative. Program representatives attended the Collaborative Launch in September 2017 to orient them to the shared aims, technical content and expectations for participation. Participating programs consented by signing a letter of understanding before joining the Collaborative. Once program representatives were familiar with the Collaborative content, they were asked to form *improvement teams* and draft team-specific aims. Teams began to collect quality indicators by creating patient registries and standardizing their clinical data entry. Teams were provided with a series of evidence-based change ideas [27] that were aligned with CCM concepts^8^ (available online here: http://stophivaids.ca/STOP/wp-content/uploads/post/BOOST-Collaborative-Change-Package.pdf). Teams selected change ideas based on their local context, expertise and patient population.

#### Support activities

In-person learning sessions were held every three to four months bringing together representatives from each team. Participants learned about quality improvement, best-practices in OUD care and the provincial response to the overdose crisis. Participants also shared their own progress and learnings and were given dedicated time to plan for future tests of change.

Learning sessions were followed by action periods where teams conducted rapid tests of change using Plan-Do-Study-Act (PDSA) cycles and completed monthly quality indicator reports along with narrative descriptions of changes. Teams were supported with monthly educational webinars, a Collaborative electronic mailing list (listerv), monthly in-practice coaching visits by the core team (medical lead, Collaborative lead and QI coach), a website of resources, and targeted monthly team feedback.

The BOOST Collaborative staff met with Vancouver Coastal Health leadership every quarter to provide progress updates. Meetings were focused on highlighting improvement processes, support needed for teams, and team reported barriers and enablers.

### Measurement and evaluation

#### Data standardization

The majority of teams participating in the BOOST Collaborative used the same electronic medical record (EMR) for patient charting that was capable of running data queries to extract aggregate QI metrics. Prior to implementing changes, teams were supported to standardize their clinical data entry through the use of an EMR form template. This form allowed the standardization of the following clinical information: International Classification of Diseases (ICD-9 version) diagnosis code, Most Responsible Provider, OAT type, daily dose, prescription start and end date, and treatment stage. The term treatment stage refers to the nature of the prescription for OAT for the client. Treatment stage can be characterized as a new start (first ever OAT prescription); re-start (OAT prescription after treatment interruption); dose increase; dose decrease; and dose unchanged.

#### Quality indicators

Teams reported monthly aggregated, non-nominal quality metrics and qualitative description of change from October 2017 to December 2018. Teams were expected to run queries in their EMR and extract the relevant quality indicators and transfer to a Microsoft Excel spreadsheet for reporting. Teams that did not use this EMR for patient charting were expected to manually extract relevant indicators and transfer to a Microsoft Excel spreadsheet for reporting. Qualitative descriptions of change were submitted on a Microsoft Word document. The rate of team participation in reporting was calculated by dividing the total number of times a team reported by 13, the total number of reporting periods during the Collaborative.

Quality indicators were based on the *cascade of OUD care* in combination with current literature and input from the multi-stakeholder expert group. Indicators included: 1) engaged in care (a documented encounter with a primary care provider using the OUD form within the last 18 months); 2) OAT access (a documented prescription for OAT); 3) active OAT (have an active, non-expired prescription for OAT); 4) retention in care > 3 months; and 5) quality of life score (PROMIS Global 10) [28]. The impact of the changes was determined by averaging monthly quality indicators and plotting arithmetic means on run charts, a common tool used for visualizing improvement. A detailed description of the analysis is forthcoming.

#### Implementation scores

From October 2017 to November 2018, teams were assigned an implementation score as a measure of progress in meeting their Collaborative aims and implementing changes. Scores ranged from 0.5 (no activity) to 5.0 (outstanding sustainable results) based on adapted Collaborative Assessment Scale criteria developed by the Institute for Health Care Improvement [29].

Implementation scores were determined by two reviewers. Monthly qualitative and quantitative reports were divided between the two reviewers and a score was applied. To ensure consistency between reviewers, the pair met monthly to review and discuss the assigned score. Final scores were applied after consensus between the two reviewers.

## Results

### Participating teams

Seventeen diverse health care teams participated in the BOOST Collaborative. Participating teams included 12 health authority owned and operated community health centres; two contracted primary health care agencies; one outreach focused team; and one rapid access SUD service and one HIV speciality clinic both located in a hospital setting. All teams were located in the Vancouver Community region (Downtown Vancouver, East Vancouver and South Vancouver). All teams were interdisciplinary with approximately five members per team and representation from key disciplines (e.g. nursing, medicine, social work and management).

Each team developed their own site-specific aim aligned with the overall Collaborative aims based on their population of focus and program mandate. Each team was encouraged to select from a series of evidence-based change ideas [27] (available online here: http://stophivaids.ca/STOP/wp-content/uploads/post/BOOST-Collaborative-Change-Package.pdf) and were supported to run rapid tests of change (using PDSA cycles) and implement the changes if they were found to be successful. Evidence-based changes to improve access to care included identifying clients lost to care (no encounter for > 6 months), improving intake forms (standardizing data entry), proactive monitoring and follow-up after missed doses of OAT, and adding reminder or follow-up calls for appointments.

### Client population

A total of 4301 patients with OUD were identified as the population of focus within the 17 participating teams. The mean age of patients was 43 (SD = 11) with 64% identifying as male, 35% female and 1% trans or non-binary. The majority of patients lived in the Vancouver region (76%) and approximately half of participants had a MRP assigned to them.

### Fidelity to BTS components

The BOOST Collaborative closely followed the IHI’s BTS Methodology, however small adaptations were accepted to support interdisciplinary participation and allow for site-specific customization. In some cases, team selection criteria were waived if a team could align itself with the overall Collaborative aims and demonstrate an ability to participate fully in the Collaborative process. For example, team selection criteria required the participation of an OAT prescriber (physician or nurse practitioner); however, this criterion was waived for an outreach-focused team made up of nurses and social workers, whose main goal was to provide outreach support following an overdose and connect those participants to care. Decisions regarding selection criteria were made by the medical lead and VCH project sponsors. In addition, all teams were supported with in-practice QI coaching, but the type and intensity of this coaching varied between teams. The adherence to the PDSA approach to testing changes also varied between teams. Finally, the 18-month timeline of the BOOST Collaborative is a modification to the traditional short-term BTS Collaborative methodology.

### Quality Indicator reporting

Teams submitted qualitative and quantitative reports electronically each month to the Collaborative staff. Median qualitative reporting rate was 42% and ranged from 15 to 85% and the median quantitative reporting rate was 35% and ranged from 0 to 77%.

### Implementation scores

Fourteen of the 17 participating teams were given a monthly implementation score. Two of the 17 teams started receiving monthly implementation score on the third month of reporting and one of the teams started receiving implementation scores on the seventh month of reporting. This delay was due to challenges and limitations in data collection and capacity.

**Fig. 2:**
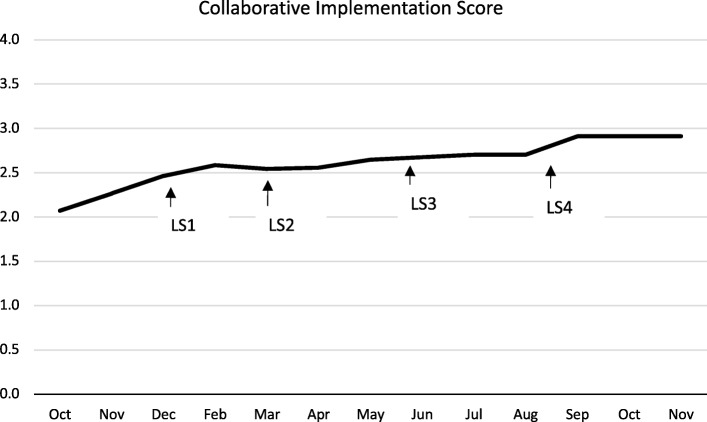
Collaborative Implementation Scores

Median implementation scores rose from 2.0 in October 2017 to 3.0 in November 2018 (Fig. 2). Data showed that 41% of teams achieved a 2.5 (changes tested, but no improvement); 35% achieved a 3.0 (modest improvement); and 24% achieved a 3.5 (improvement).

## Discussion

From July 2017 to December 2018, 17 interprofessional healthcare teams were enrolled to participate in an 18-month QI Collaborative. Participating teams made significant progress implementing evidence-based changes to clinic workflow and care processes with a focus on initiation and retention of OAT to improve outcomes for their patients with OUD. Changes to improve access to care included identifying clients lost to care, improving intake forms and adding reminder or follow-up calls for appointments. As a result, there was a significant increase in the number of individuals retained in OAT at the three-month mark from three out of ten to seven out of ten. Results from this study provide evidence to support the implementation QI and process improvement to improve retention on OAT for people living with OUD.

Improvements in care were captured in monthly reports containing process and outcome indicators and qualitative descriptions of change. Reporting rates were variable among teams with a median reporting rate of 42 and 35% for qualitative and quantitative reports, respectively. By reporting monthly on their progress, teams were able to determine if the changes they were implementing in practice, were indeed resulting in an improvement. Although reporting rates were variable among teams, this is consistent with other similar initiatives [12], and may reflect the reality that not all teams are able to establish a system of measurement to inform their quality improvement initiatives.

As part of the preparation phase for the BOOST Collaborative, the core team sought to address known issues with data quality in participating teams’ EMRs. It has been shown that data quality can significantly influence the accuracy of quality indicators [30]; therefore, we aimed to improve the fidelity of clinical data as it was entered into the EMR. EMR forms have been found to improve the likelihood of patients receiving evidence-based care and the accuracy of both clinical and administrative data [31]. This is also in line with the CCM, which highlights the importance of decision support and information systems in the proactive care of patients with chronic conditions [8].

Monthly reports were used by the core team to assign a monthly Collaborative Assessment Score [29], a characterization of team progress throughout the Collaborative process. Overall, teams showed improved progress throughout the Collaborative, with the majority of teams (59%) showing modest or some improvement. The relatively modest rates of improvement between teams may be explained by the challenges outlined above regarding data extraction and indicator reporting. Without demonstration of measurable improvement, teams cannot progress to a score greater than 2.5 (changes tested), limiting their ability to demonstrate improvements. Assessment scores do not fully capture the complexity of changes made and, when combined with quality indicator outcomes, can provide further information about team success and what may predict success for a participating team.

The BOOST Collaborative designed a QI initiative including a suite of OUD intervention strategies based on the HIV cascade of care [32]. This process requires taking a system-level view and systematically identifying gaps in care for people living with OUD from screening to diagnosis to follow-up. Further, it involves meaningfully including the client and family voice to characterize gaps in care and address this complex issue. The evidence clearly shows that people living with OUD have better health outcomes when engaged in care and retained in treatment, such as OAT [3, 4]. Retention in OAT is often suboptimal for a number of different reasons, including barriers to induction and titration, limited drop-in or clinic hours, and reactive patient recall systems. This initiative supported teams to more accurately identify and characterize patients with OUD (i.e., age, treatment stage, engagement in care and retention), and target interventions and changes based on that information to improve care and outcomes.

### Limitations and implications

Unlike other similar QI initiatives, where healthcare teams go through an application process and pay to participate [12–14], Vancouver BOOST participants were selected by health authority leadership. This may have influenced team’s receptiveness and readiness to participate in this initiative. Without an application process, there was a high variability of leadership support and commitment to quality improvement processes among teams. This may have impacted the ability of teams to engage fully in this work, particularly if time and resources were not consistently available to participants. Strong leadership commitment and financially accountable teams may have improved participants engagement in this initiative.

Although teams were provided with access to in-practice coaching and support to extract data from their EMRs, some teams had ongoing challenges with measurement and reporting and the workload it placed on staff. Qualitative data reporting rates ranged from 15 to 85% and quantitative data reporting rates ranged from 0 to 77%. The differences in reporting rates may be partially explained by variable technical skills and comfort with EMR data and functionalities. Further to this, there were ongoing and, in some cases, significant challenges with EMR data quality, which created barriers to extracting useful quality indicator data, resulting in low reporting rates for some teams. A parallel commitment from service providers, vendors or internal information technology teams to support data fidelity may have improved this element of the Collaborative.

Baseline QI knowledge and uptake of QI resources was also highly variable among teams. Some teams had participated in previous QI Collaboratives and were very familiar with QI methodology and resources, whereas others had no knowledge at all. A considerable amount of time was dedicated up front to establishing a common understanding of QI among teams. Ongoing and ad-hoc training for new staff may have improved engagement from participating teams.

Other factors that may have influenced the success of implementation and team participation included a provincial system-wide primary care redesign initiative occurring in parallel with the BOOST Collaborative. This resulted in program reorganizations and restructuring, in addition to clinic moves. This was disrupting for many of the teams as staff and caseloads were redistributed.

Finally, implementing a QI Collaborative was very challenging in the midst of a public health emergency and substantial patient mortality. For participating teams from low-barrier clinics with no set appointment times, there were challenges related to protecting time for meetings and sourcing backfill staff to attend support activities given unpredictable and fluctuating demands. This initiative offers some encouraging results for improving care for people living with OUD; however, additional research is needed to determine the sustainability of these improvements beyond the end of the Collaborative.

## Conclusions

The BOOST Collaborative developed a training program based on in-person and online learning sessions to translate evidence-informed best practices for treatment using OAT, introduce PDSA cycles for testing small changes in the clinical setting and create opportunities to share the successes and challenges of implementing these changes while striving for 95% retention on OAT. This initiative has successfully supported clinics to incorporate changes, such as adjusting clinic hours, increasing outreach capacity and structure, creating comfortable waiting rooms, engaging with pharmacies, incorporating the client/family voice as well as peers into the delivery model. Monthly qualitative and quantitative reports indicated that the majority of teams demonstrated some improvement using this intervention strategy.

Vancouver BOOST teams made significant progress in identifying areas of improvement and implementing changes with a focus on initiation of and retention on OAT. Changes to improve access to care included identifying clients lost to care, improving intake forms and adding reminder or follow-up calls for appointments. As a result, there was a significant increase in the number of individuals retained in OAT at the three-month mark. Results from this study support the feasibility of implementing an 18-month BTS quality improvement Collaborative in a publicly-funded health care system in Vancouver, Canada to improve access to care and retention on OAT.

## Data Availability

The data that support the findings of this study are available from the corresponding author, RB, upon reasonable request. However, note that participating teams only reported aggregated, non-nominal data.

## Abbreviations

BC: British Columbia
BC-CfE: B.C. Centre for Excellence in HIV/AIDS
BOOST: Best-practice in Oral Opioid agoniSt Therapy
BTS: Breakthrough series
CCM: Chronic care model
EMR: Electronic medical record
ICD-9: International Classification of Diseases
IHI: Institute for Healthcare Improvement
MRP: Most responsible provider
OAT: Opioid agonist therapy
OUD: Opioid use disorder
PDSA: Plan-Do-Study-Act
QI: Quality improvement
SD: Standard deviation
SUD: Substance Use Disorder
VCH: Vancouver Coastal Health
